# Transcription, mRNA Export, and Immune Evasion Shape the Codon Usage of Viruses

**DOI:** 10.1093/gbe/evab106

**Published:** 2021-05-14

**Authors:** Christine Mordstein, Laura Cano, Atahualpa Castillo Morales, Bethan Young, Alexander T Ho, Alan M Rice, Michael Liss, Laurence D Hurst, Grzegorz Kudla

**Affiliations:** 1 MRC Human Genetics Unit, Institute for Genetics and Molecular Medicine, The University of Edinburgh, Edinburgh, UK; 2 The Milner Centre for Evolution, Department of Biology and Biochemistry, University of Bath, Bath, UK; 3 Thermo Fisher Scientific, GENEART GmbH, Regensburg, Germany

**Keywords:** codon usage, virus evolution, gene regulation

## Abstract

The nucleotide composition, dinucleotide composition, and codon usage of many viruses differ from their hosts. These differences arise because viruses are subject to unique mutation and selection pressures that do not apply to host genomes; however, the molecular mechanisms that underlie these evolutionary forces are unclear. Here, we analyzed the patterns of codon usage in 1,520 vertebrate-infecting viruses, focusing on parameters known to be under selection and associated with gene regulation. We find that GC content, dinucleotide content, and splicing and m^6^A modification-related sequence motifs are associated with the type of genetic material (DNA or RNA), strandedness, and replication compartment of viruses. In an experimental follow-up, we find that the effects of GC content on gene expression depend on whether the genetic material is delivered to the cell as DNA or mRNA, whether it is transcribed by endogenous or exogenous RNA polymerase, and whether transcription takes place in the nucleus or cytoplasm. Our results suggest that viral codon usage cannot be explained by a simple adaptation to the codon usage of the host—instead, it reflects the combination of multiple selective and mutational pressures, including the need for efficient transcription, export, and immune evasion.

**Significance statement**

The genetic code specifies how the information in the DNA is used to make proteins. Different organisms use their DNA to encode proteins in slightly different ways, akin to people speaking different dialects across a country. Here, we investigated why viruses that infect humans use different genetic dialects, and we found that it is related to where in the cell the viruses are found, and to the different ways the viruses use to evade the immune system.

## Introduction

The universal genetic code is degenerate, with all but two amino acids encoded by two to six different codons, termed synonymous codons. Synonymous codons for a given amino acid are not equally represented among genes, and the preference for a particular set of codons in a given gene (or genome) is known as codon usage bias (CUB) (reviewed in Plotkin and Kudla (2011)). CUB can be driven by genetic drift, mutational pressure, and/or selection pressure through different molecular mechanisms (Bulmer 1991). Known mutational mechanisms include CpG methylation and deamination, nucleotide biases associated with transcription, GC-biased gene conversion, and biased DNA repair (Wolfe et al. 1989; Eyre-Walker 1993; Kaufmann and Paules 1996; Green et al. 2003). Selective pressure can be related to the altered expression level of the gene that includes the synonymous mutation (known as cis-effects of synonymous mutations), or to the metabolic cost of the mutation (trans-effects). The relative effects of mutation and selection on CUB depend on additional factors such as the expression level of the gene (Duret and Mouchiroud 1999; Francino and Ochman 2001) and the effective population size of the species (Galtier et al. 2018).

Traditionally, selection on codon usage has been studied in the context of tRNA availability and translation (Grantham et al. 1981; Gouy and Gautier 1982; Ikemura 1985; Sharp et al. 1986; Powell and Moriyama 1997; Akashi and Eyre-Walker 1998; Hershberg and Petrov 2008; Yu et al. 2015). However, in recent years, it has been recognized that codon usage correlates with a broad range of molecular phenotypes, including mRNA abundance (Kudla et al. 2006), transcription (Zhou et al. 2016; Zhou et al. 2018), splicing (Parmley et al. 2006; Julien et al. 2016), mRNA localization (Courel et al. 2019; Mordstein et al. 2020; Zuckerman et al. 2020), mRNA lifetime (Presnyak et al. 2015), RNA toxicity (Mittal et al. 2018), translation initiation (Kudla et al. 2009; Goodman et al. 2013; Cambray et al. 2018), and protein folding (Kimchi-Sarfaty et al. 2007; Zhou et al. 2013; Buhr et al. 2016; Walsh et al. 2020). These phenotypic effects are not necessarily linked to the usage of synonymous codons per se, but rather to properties of the coding sequence that covary with codon composition: RNA folding energy, GC content, CpG content, and the presence of certain sequence motifs such as exonic splice enhancers (ESEs) or RNA modification sites. In the discussion below, we will for simplicity use the term CUB to refer to any deviation from random usage of synonymous codons, whatever the underlying mechanism might be.

It has long been debated whether the codon usage of viruses should resemble the codon usage of their hosts (Bahir et al. 2009; Wong et al. 2010; Butt et al. 2014; Nasrullah et al. 2015; Kumar et al. 2016). In a broad study of RNA and DNA viruses that infect hosts ranging from bacteria to human, Bahir et al. (2009) reported that host mimicry is not consistent across viruses with different host species. For example, viruses infecting bacteria show a similar GC content to their hosts, but this is not true for mammalian-infecting viruses. Vertebrate RNA viruses present a wide range of GC contents, from 33% (respiratory syncytial virus) to 70% (rubella virus), and the mechanisms underlying this variation are still elusive (Odon et al. 2019). Several hundred species of viruses are known to infect humans. These viruses differ in the type of genetic material they carry (RNA or DNA, single- or double-stranded, and positive or negative strand); genome structure (segmented or nonsegmented), capsid shape, presence or absence of an envelope, target tissue for primary infection in host; mechanism and location of replication in the host cell (Hulo et al. 2011); and specialized mechanisms to avoid detection and inactivate antiviral response pathways in the host. Unsurprisingly, viruses with different life strategies are under unique mutational and selective pressures that influence their CUB. For example, viruses that target different tissues show different patterns of tRNA adaptation (Hernandez-Alias et al. 2021); viruses that are transcribed in the nucleus, but not in the cytoplasm, are under pressure to evolve sequence elements that prevent mis-splicing, because the splicing machinery is primarily found in the nucleus. At the same time, binding of splicing factors may be required for facilitating efficient expression, analogous to the observed positive selection on ESE motifs in intronless human genes (Savisaar and Hurst 2016). Similar selective pressures have been observed for internal RNA modifications such as *N^6^*-methyladenosine (m^6^A). Besides its role in splicing (Ye et al. 2017), stability (Wang et al. 2014), translation (Meyer et al. 2015; Wang et al. 2015) and replication of viral RNA (Manners et al. 2019), m^6^A methylation of virus RNA helps to escape a RIG-I dependent interferon-I response by the host (Lu et al. 2020), a strategy that was later shown to be conserved across several RNA virus families (Bayoumi and Munir 2021). Both ESE motifs and m^6^A motifs constitute means to mark viral RNA as “self” thereby circumventing host innate immunity and allowing efficient virus expression. In contrast, viruses that have mechanisms to evade and shut off the host immune response early during infection are under lower pressure to avoid recognition by innate immune sensors (Lin et al. 2020).

Despite many years of study, fundamental questions remain regarding the codon usage of viruses. Does viral codon usage reflect primarily an adaptation of the virus to its cellular environment, or mutational processes directed by the host to fight the virus? How can viral genes be efficiently expressed, given the large differences of codon and nucleotide composition from their hosts? Why are there closely related viruses with very different codon usage?

To address these questions, we assembled a comprehensive database of protein-coding sequences of vertebrate-infecting viruses and analyzed their codon usage. Having observed correlations between viral replication compartment (cytoplasm or nucleus) and codon usage, we designed an experimental model based on T7 phage RNA polymerase-driven transcription to study the effects of transcription compartment in gene regulation. The results of our analyses and experiments point to a role of transcription and nuclear export of mRNA in shaping the codon usage and expression of viral genes.

## Results

With notable exceptions (Bahir et al. 2009), most previous analyses of nucleotide composition and codon usage biases in viruses were limited to individual species or subgroups, such as RNA or DNA viruses (Jenkins and Holmes 2003; Shackelton et al. 2006; Belalov and Lukashev 2013; Kustin and Stern 2021). In order to investigate codon usage across a large set of vertebrate-infecting viruses, we collated information from multiple sources (see Methods) to assemble a database of protein-coding sequences from 1,520 virus species belonging to 48 families, including most viruses known to infect humans (supplementary table 4, Supplementary Material online). Unlike genes in higher organisms, many open reading frames in viruses either overlap or are contained within others. Such dual-coding regions are likely subject to selection on amino acid sequence in both frames, potentially confounding analyses of codon usage. To mitigate this, we employed two levels of filtering: First, cases in which coding sequences are contained within another coding sequence were both excluded (i.e., both internal, as well as surrounding coding sequence). Second, for partially overlapping coding sequences, we adjusted coordinates to remove the overlapping portions of each gene. All adjustments were made in increments of 3 nt to retain the correct reading frame in the remaining unique sequence portions.

**Fig. 1.**
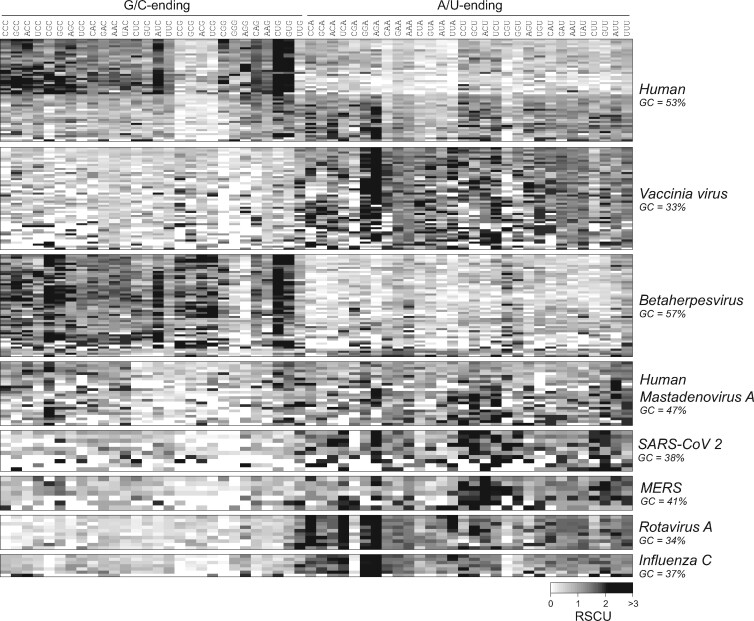
Codon usage of virus and human genes. The relative synonymous codon usage (RSCU) is shown for 50 random human genes and 7 vertebrate-infecting viruses. Values range from 0 (the codon is absent) to 1 (no bias) to 6 (only one codon is used in a six-codon family).

Figure 1 shows the codon usage in human and selected viruses, with each horizontal line representing the codon usage of a human gene (50 randomly selected genes), or a gene from a human-infecting virus. Several patterns are apparent: human genes differ from each other in their codon usage, with approximately half of the genes preferring C- and G-ending codons (left side of the heatmap), and others showing no codon preference or preferring A- and U-ending codons. CpG-ending codons and, to a lesser degree, UpA-ending codons are depleted in most human genes. The CUG codon is enriched in human, while CGC and AGA codons are strongly enriched in some genes but depleted in others. By comparison, codon usage is relatively uniform within individual viruses but varies remarkably between viruses. Many viruses have a strong preference for A- and U-ending codons (e.g., vaccinia, rotavirus, influenza viruses, papillomaviruses, coronaviruses, and some retroviruses including HIV), while others prefer C- and G-ending codons (some herpesviruses, adenoviruses, and retroviruses). The depletion of CpG- and UpA-ending codons is seen in some, but not all viruses. Some viruses show unique preferences of specific codons, for example, UCA is strongly enriched while CGA is depleted in rotavirus A, even though both codons show an intermediate frequency in human.

**Fig. 2.**
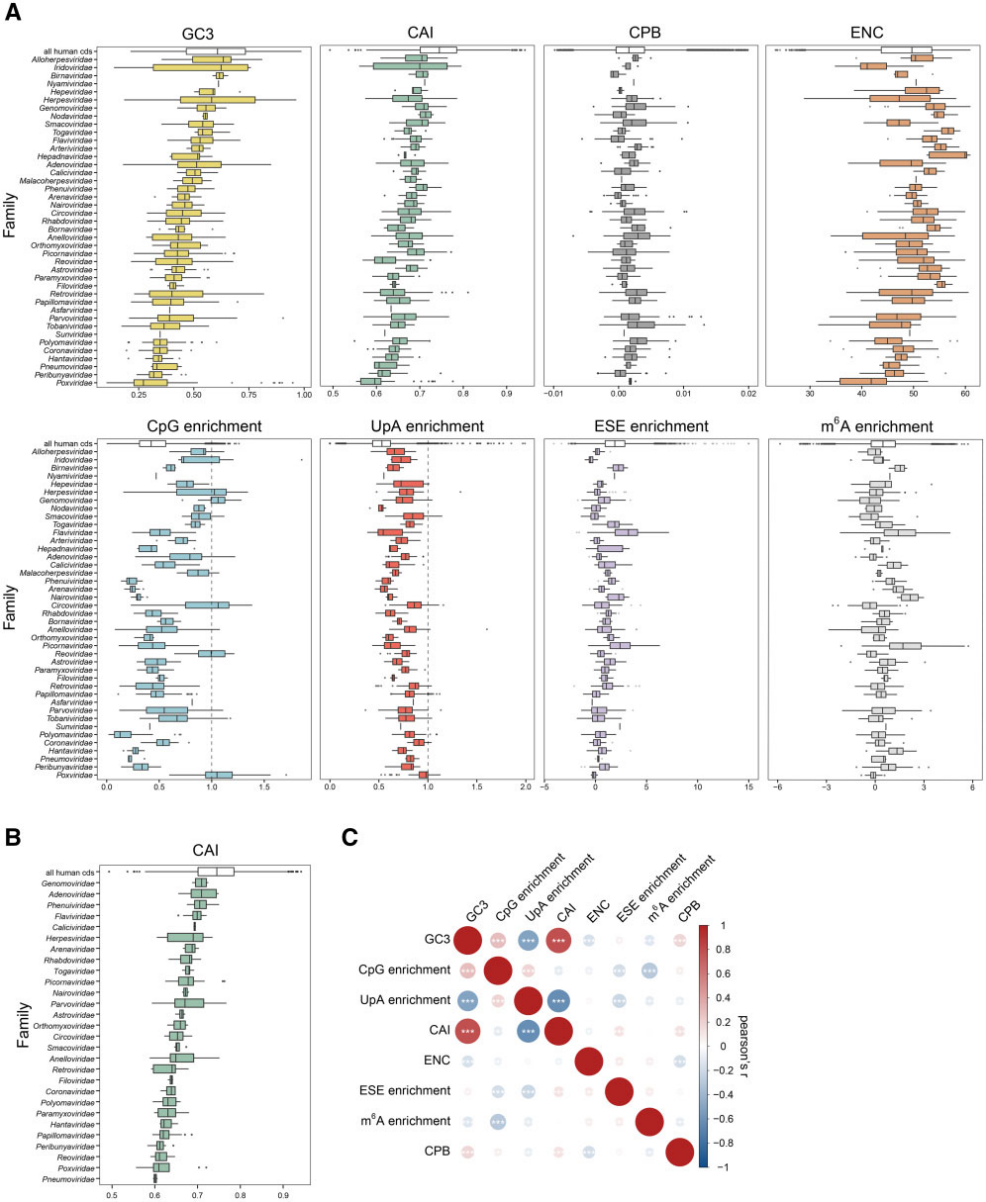
Comparison of sequence-derived parameters of vertebrate-infecting virus genes. Commonly used measures of codon usage bias were calculated for all virus genes, averaged per virus species and grouped by family. (A) Boxplot representation of the variation in sequence features within each of 41 virus families. For comparison, the same parameters were calculated for human coding genes (top-most white boxes; only the longest transcript isoform per gene was used; *n* = 20,075). Shown are the relative G- and C-nucleotide content at third codon sites (GC3), the codon adaptation index (CAI), codon pair bias (CPB) and effective number of codons (ENC). The CpG and UpA dinucleotide enrichment are calculated as described in Materials and Methods: a value <1 indicates dinucleotide avoidance, while >1 indicates enrichment. Additionally, splicing-related features, such as the enrichment of exonic splice enhancer (ESE) motifs and enrichment of N^6^-methyladenosine consensus sequence are shown. See also supplementary figure S1, Supplementary Material online and Materials and Methods. (B) Boxplot representation of the variation in CAI for species of human-infecting viruses only. (C) Pearson correlation matrix of all sequence parameters shown in (A).

As hinted above, patterns of codon usage are not necessarily caused by translational preferences for specific codons, but they may be driven by mutational or selective pressures on nucleotide composition or on sequence motifs with diverse biological functions. To evaluate the variation of these properties across virus families, we calculated the distributions of selected sequence-derived parameters among human and viral genes (fig. 2). Strikingly, almost every virus family differed significantly from human genes in most parameters (fig. 2*A*, supplementary table 1, Supplementary Material online). The human codon adaptation index (CAI) measures the similarity between the codon usage of a gene and a set of highly expressed human genes, and we found that as a group, human genes had higher CAI than almost all families of viruses (36/41), even though the highly expressed human genes used to define CAI were excluded from this comparison. This was also true when the analysis was limited to viruses known to infect humans, rather than all vertebrate viruses (fig. 2*B*, supplementary table 1, Supplementary Material online). Human genes were also more GC-rich (32/41), more enriched in ESE sequence elements (31/41), and less enriched in UpA dinucleotides (34/41) than almost all families of viruses. By contrast, viruses were highly variable in their CpG contents, with families such as *Phenuiviridae* or *Polyomaviridae* being more depleted in CpG than human genes, while others, such as *Reoviridae* or *Poxviridae*, showing no CpG depletion. Many virus families also differed from human genes in their codon pair bias (CPB; 14/41), m^6^A motif enrichment (24/41), and effective number of codons (ENC; 27/41). Even though the ENC is sometimes used as a proxy to compare the codon usage between viruses and their hosts (Sheikh et al. 2020), similarity in ENC does not indicate an overall similarity of codon usage. For example, *Coronaviridae* have a similar distribution of ENC to human genes, but they differ significantly from human genes in all other parameters we analyzed except CPB. This is perhaps not surprising, given the lack of correlation between ENC and other descriptors of codon usage (fig. 2*C*). Similar results were obtained when analyzing the codon usage of virus families on a per-species level (fig. 2) and per-gene level (supplementary fig. S1, Supplementary Material online). Extreme differences of GC content, CpG, CAI, and other sequence properties were also apparent between closely related viruses belonging to the same family, such as *Herpesviridae*, *Poxviridae*, or *Adenoviridae* (fig. 2, supplementary fig. S2, Supplementary Material online). Taken together, these results indicate that human-infecting viruses do not mimic the codon usage of human genes, even though they may appear to do so when focusing on specific descriptors of codon usage.

**Fig. 3.**
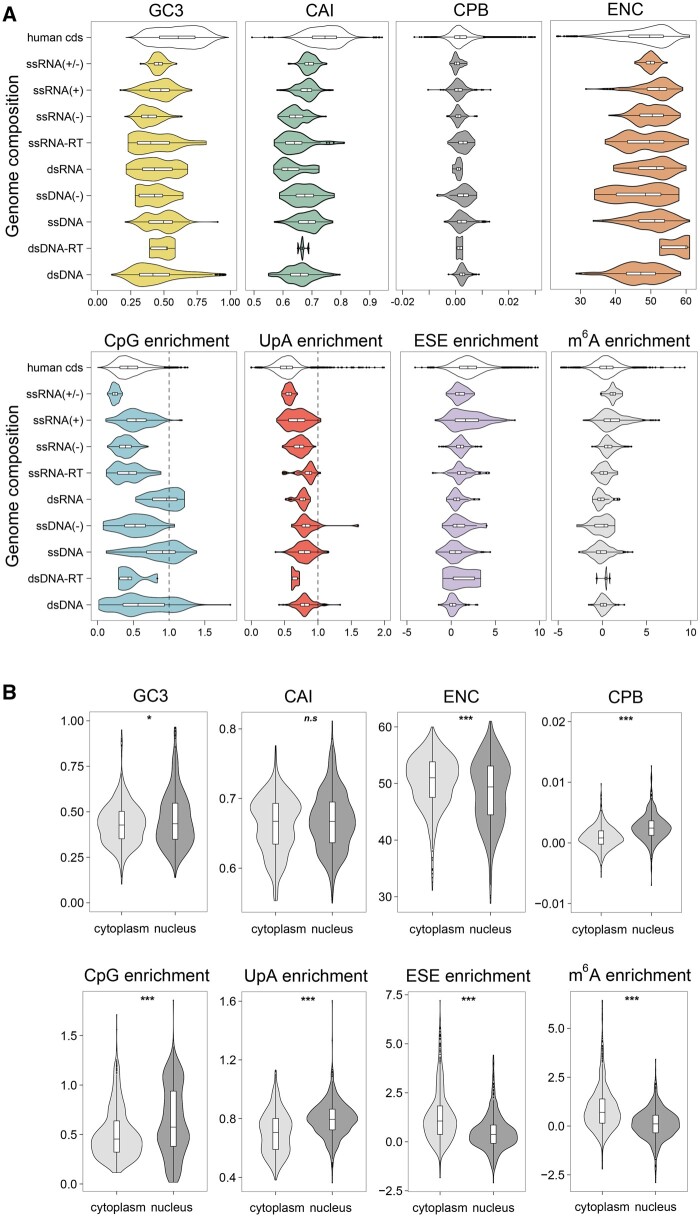
Associations of sequence features with virus properties. Comparison of sequence-derived parameters of virus genes depending on (A) genome composition according to the Baltimore classification (Baltimore 1971), or (B) subcellular compartment of virus transcription (Hulo et al. 2011).

Previous studies showed associations between the codon usage of viruses and properties such as genetic material (DNA or RNA), genome replication compartment (nucleus or cytoplasm), or duration of infection (acute or persistent) (Chen 2013; Jitobaom et al. 2020). In addition, viruses that infect vertebrates and invertebrates (vector-borne) might be under different mutational and selective constraints, compared to those that only infect vertebrate hosts. To study these associations in more detail, we grouped viral genomes by their composition according to the Baltimore classification (Baltimore 1971) as defined by the International Committee on Taxonomy of Viruses (ICTV) (Lefkowitz et al. 2018). As reported previously (Rice et al. 2020), DNA viruses were typically more GC-rich than RNA viruses, but GC content also depended on the strandedness of the genetic material, with ssDNA(+) and ssRNA(+) viruses showing higher GC contents than ssDNA(−) and ssRNA(−) viruses, respectively (fig. 3*A*). Although most RNA viruses were strongly depleted of CpG dinucleotides, dsRNA viruses were not, whereas ssDNA and dsDNA viruses showed broad distributions of CpG content, ranging from near-total CpG depletion in some species to moderate CpG enrichment in others. RNA viruses tended to show the highest relative enrichment of ESE and m^6^A motifs, with most ESE motifs found in ssRNA(+) viruses, and most m^6^A motifs in ssRNA(+) and ssRNA(+/−) species. Codon usage also depended on the replication compartment, with viruses replicating in the nucleus being more GC-rich, CpG-rich, and UpA-rich, but showing lower ESE and m^6^A scores, compared to viruses that replicate in the cytoplasm (fig. 3*B*, supplementary fig. S4, Supplementary Material online). The degrees of CpG and UpA enrichment were correlated with each other in cytoplasmic viruses (*r* = 0.32, *P* = 5.6 × 10^−19^), showing that selection for (or against) CpG tends to coincide with selection for or against UpA in these species (supplementary fig. S4, Supplementary Material online). In addition, CpG and UpA depletion were more pronounced in linear or segmented genomes, compared to circular or monopartite genomes (supplementary fig. S3, Supplementary Material online). Notably, the only parameter that did not differ between nuclear and cytoplasmic viruses was CAI, possibly indicating that nuclear and cytoplasmic viruses are under a similar pressure to match their codon usage to the translation machinery of their host.

**Fig. 4.**
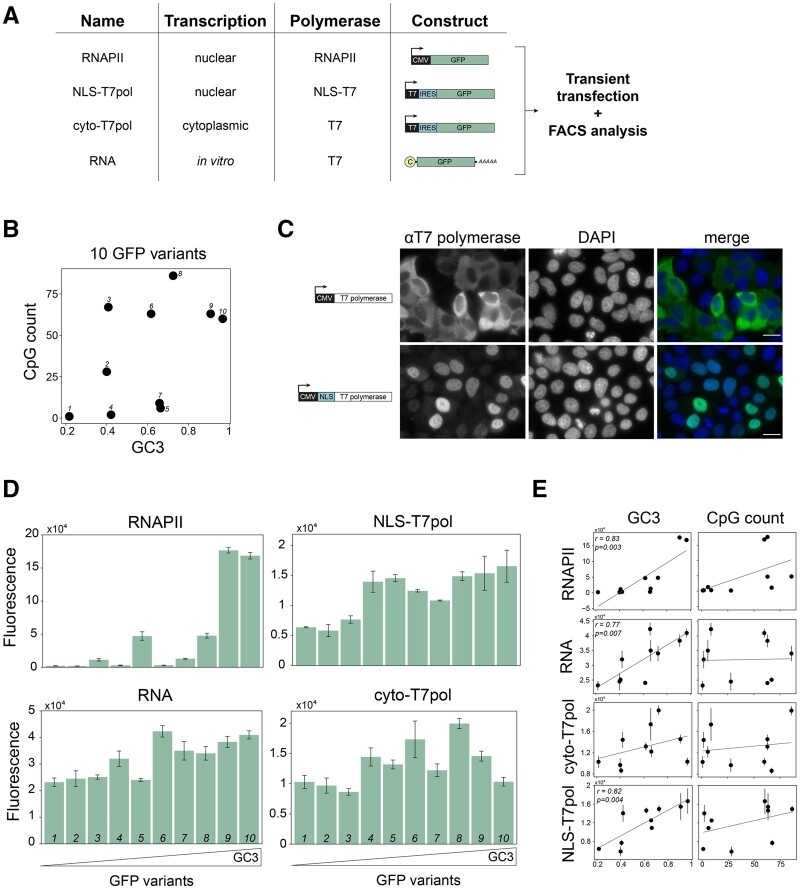
Effects of codon usage on gene expression depend on transcription machinery and subcellular compartment. (A) Schematic outline of experimental system to compare effects of codon usage on gene expression depending on transcription machinery and subcellular localization. (B) GC3 and CpG content variation of 10 synonymous GFP variants tested in (A). Variants are numbered from 1 to 10 by increasing GC3. (C) Immunofluorescence staining of HeLa cells transiently expressing either T7 polymerase (top row, left panel) or T7 polymerase with a nuclear localization signal (NLS; bottom row, left panel). Nuclei were stained with DAPI. Scale bar = 20 μm. (D) GFP expression as measured by Fluorescence-activated cell sorting (FACS) of HeLa cells expressing different GFP variants transcribed by either endogenous RNA polymerase II (RNAPII), cytoplasmic (T7) or nuclear T7 polymerase (NLS-T7), or transfected with in vitro synthesized RNA. The order of GFP variants is arranged as described in (B). Each data point represents the mean of 3 independent replicates ± SEM. (E) Correlations of GC3 and CpG count with GFP expression in all measured expression systems.

Given the variation of codon usage among human viruses, and its association with the genome composition and subcellular compartment of genome replication and transcription, we designed an experiment to test whether codon usage might have different effects on gene expression depending on how the genetic material is delivered to the cell. To answer this question, we measured the expression of synonymous reporter genes in four heterologous expression systems (fig. 4*A*): 1) a standard mammalian expression system (referred to as “RNAPII” in the figure), in which the reporter gene is placed in a DNA plasmid under the control of a CMV promoter and is transcribed in the cell nucleus by endogenous RNA polymerase II; 2) an mRNA transfection system (RNA), in which capped and polyadenylated mRNA is transcribed in vitro by phage T7 RNA polymerase (T7 RNAP) and transfected into cells; 3) a nuclear T7 transcription system (NLS-T7 Pol), in which the reporter gene is placed in a DNA plasmid under the control of a T7 RNAP promoter and an internal ribosome entry site (IRES), and is cotransfected into cells together with a nuclear-restricted version of T7 RNAP; and 4) a cytoplasmic T7 transcription system (cyto-T7 Pol), which differs from the above by using a cytoplasmic-restricted version of T7 RNAP. The two versions of T7 RNAP differed by the presence of a nuclear localization signal in one of the constructs, and we verified by immunofluorescence staining that each T7 RNAP variant was directed to the intended compartment (fig. 4*C*). We also verified that the expression of reporter genes from the T7 promoter constructs increased >10-fold upon cotransfection of T7 RNAP, indicating *bona fide* T7-driven transcription within cells (supplementary fig. S5, Supplementary Material online).

To analyze the effects of codon usage in the four expression systems, we used 10 synonymous variants of the GFP gene that all encoded the same protein sequence but varied in codon usage (fig. 4*B*). Our GFP variants covered most of the range of GC content and CpG enrichment observed in viruses, and they also varied in other sequence properties that correlate with codon usage (supplementary table 2, Supplementary Material online). When the variants were expressed using the RNAPII system, we observed 95-fold variation in expression, with the GC-poor variants expressed at near-background levels and efficient expression of GC-rich variants (*P* = 0.003, *r* = 0.83, fig. 4*D* and *E*), as seen previously (Kudla et al. 2006; Mordstein et al. 2020). Four out of five variants with high CpG content were highly expressed in the RNAPII system, but it was difficult to determine if this was an independent effect, or if it resulted from the covariation between GC content and CpG content in our constructs (fig. 4*B*). Surprisingly, when the GFP variants were expressed in the RNA transfection or T7 systems, the range of variation was much smaller, with no more than 3-fold difference between the lowest and highest-expressed constructs (fig. 4*D* and *E*). High GC content was significantly associated with increased expression in the RNA system (*P *= 0.007, *r* = 0.77), and in the NLS-T7 Pol system (*P *= 0.004, *r* = 0.82), but not in the cyto-T7 Pol system (*P* > 0.05). CpG content was not associated with expression in any system. Taken together, these results show that the effects of GC content on gene expression depend on whether the genetic material is delivered to the cell as DNA or RNA, whether it is transcribed by endogenous or exogenous RNA polymerase, and whether transcription takes place in the nucleus or cytoplasm.

## Discussion

As in free-living organisms, the codon usage of viruses is the outcome of genetic drift, mutational pressure, and/or selection pressure (Bulmer 1991). Selection drives viral codon usage towards efficient utilization of the host tRNA pool, production of stable RNA, avoidance of mis-splicing, utilization of host nuclear export mechanisms, and immune evasion. Mutations result from inaccurate viral replication and host mechanisms that introduce mutations in viral genomes. The combination of these pressures results in the large diversity of codon usage observed across human-infecting viruses.

### Codon Adaptation to Host

It has been reported that the codon usage of viruses is adapted to their hosts to match the availability of anticodons in the host tRNA pool (Gingold et al. 2014; Goodarzi et al. 2016; Hanson and Coller 2018). This is supported by the finding that host resemblance is not consistent throughout a virus genome and is generally stronger in structural proteins than in nonstructural proteins (Kazazian 2004; Bahir et al. 2009). Recently, Chen et al. (2020) suggested that if the codon usage of a virus is too closely matched to the host cell, the increased translational load could be detrimental, and they proposed that an intermediate level of codon adaptation should be optimal. However, even though codon adaptation to the host is expected to improve the utilization of ribosomes, and despite the large population sizes of viruses which should in theory facilitate selection for well-adapted codons, we observed that the CAI of most human-infecting viruses is lower than that of human genes. Selection on virus codon usage is also influenced by the translational shutoff of the host, which is induced by many viruses and which leads to large changes in demand for specific tRNAs. As a result, translationally optimal codon usage may be different for different viruses.

### Transcription and Nuclear Export

It has long been known that viruses show wider ranges of GC contents than their hosts, including mammals (Wyatt 1952; Bronson and Anderson 1994). This is especially true for families of dsDNA viruses such as *Herpesviridae*, *Adenoviridae*, or *Poxviridae* (fig. 2). The site of replication is an important determinant of viral codon usage, and the GC, CpG, and UpA contents of nuclear viruses tend to be higher than in cytoplasmic viruses (Shackelton et al. 2006; Rice et al. 2020) (see also fig. 3*B*). Most DNA viruses (except for *Poxviridae*, *Asfarviridae*, and *Iridoviridae*) replicate in the nucleus (Shackelton et al. 2006), whereas most RNA viruses (except the *Orthomyxoviridae* family which includes the influenza viruses) do not enter the nucleus, and carry out their complete lifecycle in the host cell cytoplasm. Accordingly, DNA viruses have, typically, higher GC contents than RNA viruses.

It is plausible that the higher GC content of viruses that replicate in the nucleus reflects selection for efficient nuclear export of RNA: high GC content promotes RNA export and can enhance the expression of intronless mRNAs (Mordstein et al. 2020); cytoplasmic viruses can afford to have lower GC contents as nuclear export is not required. Consistently, our experiments show a strong correlation between GC content and expression for genes transcribed by the endogenous RNA polymerase, and a moderate correlation for genes transcribed by T7 polymerase in the nucleus, but no effect of GC content on expression for genes transcribed by the T7 polymerase in the cytoplasm. Although T7 polymerase-based transcription does not necessarily represent transcription by nonphage viral polymerases, a recent study supports the modulation of codon usage effects by transcription (Yang et al. 2021).

In eukaryotes, nuclear export of mature mRNA transcripts is carried out through several pathways, which depend on characteristics such as nucleotide composition, RNA length, RNA structure, and the presence of introns and specific structural or sequence elements (for review, Masuyama et al. 2004). These export pathways are routinely exploited by viruses (Saphire et al. 2000; Ozawa et al. 2007). Recently, Ulitsky and colleagues studied the effect of transcript characteristics on the utilization of nuclear RNA export factor 1 (NXF1) and TREX export pathways. The NXF1 pathway facilitates the export of single-exon transcripts and transcripts with long exons, and upon NXF1 depletion these transcripts are retained in the nucleus (Zuckerman et al. 2020). These single-exon transcripts are AU-rich and contain conserved structural regions which drive NXF1-dependent export. Conversely, TREX preferentially exports GC-rich and spliced transcripts. Thus, nucleotide preferences of viruses might reflect the availability of specific nuclear export pathways during infection.

### Host Immune System Evasion

Mammalian cells produce pathogen recognition receptors that bind to specific molecular patterns in viral genomes, such as double-stranded RNA (dsRNA), or CpG and UpA dinucleotides (Karlin et al. 1994; Rima and McFerran 1997; Simmonds et al. 2013; Takata et al. 2017; Lin et al. 2020). These immune defences drive virus codon usage away from sequences detected by the host. As a result, suppression of CpG and UpA dinucleotides has been reported for most vertebrate RNA viruses and small DNA viruses (Karlin et al. 1994; Rima and McFerran 1997; Simmonds et al. 2013). Strangely, even though dsRNA is recognized by the host innate immune system, many viruses, including SARS-CoV-2, show unexpectedly strong secondary structures in their RNA (Forsdyke 1995; Simmonds et al. 2004; Simmonds 2020).

The recognition of CpG dinucleotides is mediated by the Zinc-Antiviral Protein (ZAP) pathway, which identifies high CpG transcripts during viral infection and restricts replication (Gao et al. 2002; Takata et al. 2017; Lin et al. 2020). Our experiments show no significant correlation between CpG content and expression, possibly reflecting low levels of ZAP activity in cells in the absence of virus infection. Despite the common depletion of CpG dinucleotides in viral genomes, it is interesting to note that many viruses do not show CpG suppression, suggesting that they may have evolved specific mechanisms to avoid detection by ZAP (fig. 2). A striking example is that of the *Herpesviridae* family, which can be divided into three subfamilies: *Alpha-*, *Beta-*, and *Gammaherpesvirinae*, all characterized by different CpG frequencies. Very recently, it has been shown that the human cytomegalovirus (*CMV*, a betaherpesvirus) only shows CpG suppression in its immediate-early transcripts, which allows evasion of ZAP and eliminates the need for CpG suppression in late expressed CMV genes (Lin et al. 2020). Another recent, and so far unexplained, finding is the CpG suppression found in all SARS-CoV-2 genes except the E and ORF-10 genes (Digard et al. 2020).

In addition to CpG, other nucleotides and dinucleotides in RNA may also be recognized by a range of immune sensors. The OAS/RNAse L system selectively targets and inhibits translation or degrades viral mRNA by recognizing UpA and UpU dinucleotides. TLR3, TLR7, and TLR8 receptors recognize unmodified nucleotides in RNA but the recognition is ablated by incorporation of modified nucleosides, such as m^5^C, m^6^A, m^5^U, s^2^U, or pseudouridine (Heil et al. 2004; Kariko et al. 2005), a finding which has been used in a spectacular way in the development of mRNA therapies and vaccines against SARS-CoV-2 (Mulligan et al. 2020). Studies increasing CpG and UpA dinucleotide frequencies in viral genomes show decreased viral infectivity and replication compared with wildtype viruses (Atkinson et al. 2014; Takata et al. 2017). The depletion of UpA and CpG dinucleotides in viruses transcribed in the cytoplasm could be explained as a mechanism to avoid host immune response, with cytoplasmic ZAP or OAS3/RNaseL attenuating viruses with increased CpG and UpA frequencies (Odon et al. 2019). These mechanisms certainly play important roles in the evolution of nucleotide preferences in viruses.

The infection by some viruses is accompanied by the production of dsRNA, which is recognized by RIG-I-like receptors (RLRs) in the cytoplasm of infected cells and triggers the type I interferon response and expression of proinflammatory cytokines. dsRNAs are also recognized by PKR, which results in a translational shut-off of the host. The remarkable differences in the strength of RNA folding between related viruses (Simmonds et al. 2004) might be accompanied by different responses to the host dsRNA-sensing systems.

### Splicing and RNA Modifications in Viruses

Another way for the host to distinguish “self” from non-selftranscripts is RNA splicing. The vast majority of virus transcripts do not contain introns and are therefore less likely decorated with factors that facilitate expression, such as SR-proteins or the Exon-junction complex (EJC). Human intronless genes have been shown to have higher GC content and higher densities of ESE motifs than expected (Savisaar and Hurst 2016), possibly to avoid detection of these transcripts as foreign by innate immune sensors. The same idea can be applied to other marks of self such as internal RNA modifications, most of which are primarily deposited in the nucleus. Recently, m^6^A has emerged as an important modulator of the host immune response as well as regulator of viral gene expression and replication by protecting viral RNAs from detection by PRRs (Brocard et al. 2017; Ye et al. 2017; Manners et al. 2019; Lu et al. 2020, 2021; McFadden et al. 2021). In the case of m^6^A, it is deposited cotranscriptionally (Slobodin et al. 2017; Louloupi et al. 2018; Zhou et al. 2019) and has been shown to have an important role in regulating alternative splicing (Xiao et al. 2016; Zhou et al. 2019), RNA stability (Wang et al. 2014; Du et al. 2016), RNA export (Roundtree et al. 2017), and translation (Meyer et al. 2015; Wang et al. 2015; Zhou et al. 2015).

Although few viruses are spliced, there is increasing evidence that those that are spliced contain functionally important splicing regulatory sequences and RNA modifications. The best-studied examples of ESEs regulating viral splicing are the M and NS segments of influenza A (Dubois et al. 2014; Huang et al. 2017), which are essential for efficient infection (Chua et al. 2013). In the case of NS1/NEP splicing, splicing efficiency is low and acts as a molecular timer of infection: NEP splicing depends partly on NS1 concentrations; increasing splice efficiency leads to virus attenuation. It was also shown recently that m^6^A is required for efficient splicing of Adenovirus (Price et al. 2020) and KSHV (Ye et al. 2017). Somewhat counterintuitively, we find that nuclear viruses have lower ESE and m^6^A scores than cytoplasmic viruses, despite the fact that many known functions of these sequence elements take place in the nucleus. It might be the case that nuclear viruses are under pressure to avoid ESE or m^6^A motifs to avoid missplicing whereas cytoplasmic viruses are under no such pressure. In the case of some cytoplasmic replicating RNA viruses, it was recently shown that cytoplasmic deposition of m^6^A can be utilized as an avoidance mechanism for recognition by host innate immunity (Lu et al. 2020, 2021) suggesting m^6^A motifs to be at least under partial selection.

In conclusion, our analyses show diverse codon usage among human-infecting viruses, and little evidence for simple adaptation of codon usage to the codon usage of the host. Rather, the codon usage of viruses reflects the combination of multiple selective and mutational pressures, including the need for efficient transcription, export, and immune evasion. At the same time, our experiments show that in contrast to endogenous RNA polymerase II transcription, transfected mRNAs and genes transcribed by T7 polymerase in the nucleus or cytoplasm of cells are surprisingly robust to changes in codon usage, which helps explain how viruses could have evolved the large diversity of codon usage we observe in nature.

## Materials and Methods

### Genes and Plasmids

Sequences of T7 polymerase and NLS-T7 polymerase were provided by Ella Sklan (Tel Aviv University, Dukhovny et al. 2018; see supplementary table 3, Supplementary Material online) and ordered pre-cloned in pcDNA3.4 TOPO from GeneArt/ThermoFisher (pcDNA3.4-T7pol and pcDNA3.4-NLS-T7pol). pUC19-T7 pro-IRES-EGFP was a gift from Fei Chen (Addgene plasmid # 138586; http://n2t.net/addgene:138586; RRID: Addgene_138586) and was further modified to resemble the wild-type ECMV IRES sequence as described by Bochkov and Palmenberg (2006; A7 was changed to A6; native *MscI* site was retained). Furthermore, *BamHI* and *EcoRI* sites outside the IRES sequence were removed and inserted downstream of the IRES to allow convenient subcloning of synonymous GFP variants from pGK3, a Gateway-compatible entry vector (Kudla et al. 2009; list of GFP variants and their sequence features in supplementary table 2, Supplementary Material online). To implement all plasmid changes, a double-stranded DNA fragment containing the above modifications was ordered from GeneArt/ThermoFisher and cloned into pUC19-T7-pro-IRES-EGFP using the unique *Kas*I and *Pac*I restriction sites. For GFP expression via endogenous RNA polymerase II, GFPs were subcloned from pGK3 into pCM1, a CMV-driven Gateway destination plasmid (Mordstein et al. 2020).

#### In Vitro RNA Synthesis

Templates for in vitro transcription were generated by amplifying the GFP sequences from pCM1 using primers T7_UTR_F: TAATACGACTCACTATAGGCTAGCCTCG and SV40_UTR_R: TGTTGTTAACTTGTTTATTGCAGCTTA. The amplicon contains part of the 5’UTR as well as the 3′UTR for increased transcript stability when transfected into cells. RNA synthesis was performed using the HiScribe T7 ARCA mRNA Kit (with tailing) (NEB, E2060S) according to the manufacturer’s instructions. Final RNA products contain the antireverse cap analog (ARCA) and a poly(A) tail. RNA was cleaned up using the Monarch RNA clean-up kit (NEB, T2040S) and RNA integrity confirmed on a Agilent Bioanalyzer 2100 using a RNA 6000 nano chip.

### Transient Plasmid and RNA Transfections

HeLa cells were grown to 70% confluency in 12-well plates in phenol-red free DMEM (FluoroBrite, Gibco) supplemented with 10% FCS and 5 mM L-Glutamine. Cotransfections of pcDNA3.4-T7pol or pcDNA3.4-NLS-T7pol with pUC19-T7pro-IRES-GFP were performed at 3:1 ratio with a total of 1ug DNA. For single plasmid controls, the respective proportion was transfected, that is, 250 ng of IRES-GFP constructs or 750 ng of T7 plasmids. For RNA transfections, 1 μg RNA was transfected per well. In brief, respective amounts of DNA or RNA were diluted in 100 μl serum-reduced OptiMEM media (Gibco). Three microliters Lipofectamine 2000 (ThermoFisher) were diluted in 100 μl OptiMEM media (Gibco) and incubated for 5 min before mixing with DNA(RNA)/OptiMEM mix. After a further 20 min, the transfection-complex was added dropwise to the cells.

#### FACS Analysis and Data Processing

Cells were trypsinized and resuspended in phenol-red free DMEM (Fluorobrite) followed by FACS analysis on a BD LSRFortessa. Cells transfected with plasmids were analyzed after 24 h, while cells transfected with RNA were analyzed 6 h post-transfection. Gating on FSC and SSC were set on viable, single cells. Gating on GFP positive cells, as well as voltage were kept constant throughout all replicate experiments to allow direct comparison. Data analysis was performed on ∼50,000 cells per sample. GFP expression was calculated as the mean fluorescence across the viable sample population. To correct for autofluorescence, the mean GFP fluorescence of the negative sample (mock-transfected cells) was subtracted from all other samples. For samples cotransfected with IRES-GFP and T7 (or NLS-T7) polymerase, the background fluorescence of single-transfections with the IRES-GFP plasmids was additionally subtracted (see also supplementary fig. S5, Supplementary Material online). FACS data can be found in supplementary table 2, Supplementary Material online.

#### Immunofluorescence Staining

200,000 HeLa cells were seeded on coverslips in 6-well plates. After 24 h, cells were transfected with 2 μg pcDNA3.4-T7pol or pcDNA3.4-NLS-T7 using 5 μl Lipofectamine. Twelve hours post-transfection, cells were fixed in 4% Paraformaldehyde/PBS for 10 min on a rocking platform, followed by 3 × 5 min washes with PBS. Cells were permeabilized with 0.5% Triton X-100/PBS for 30 min, followed by 3 × 5 min washes with PBS. Samples were blocked with 1%BSA, 0.01%Triton- X-100 in PBS for 1 h before incubating with anti-T7 polymerase antibody (Creative Diagnostics, CABT-B8990) at 1:100 in blocking buffer for 1 h. After incubation, cells were washed 3 × 5 min with PBS before incubating with 1:1,000 alexa488 antirabbit secondary (Life technologies) in blocking buffer for 1 h followed by a further 3 × 5 min washes with PBS. Nuclei were stained using 50 μg/ml DAPI solution (4′,6-diamidino-2-phenylindole) for 5 min followed by 2 × quick washes with PBS. Coverslips were mounted on microscope slides using Vectashield (Vectorlabs, H-1000-10). Epifluorescent images were acquired using a Photometrics Coolsnap HQ2 CCD camera and a Zeiss Axioplan II fluorescence microscope with Plan-neofluar objectives (63×, Carl Zeiss, Cambridge, UK), a Mercury Halide fluorescent light source (Exfo Excite 120, Excelitas Technologies) and Chroma #83000 triple bandpass filter set (Chroma Technology Corp., Rockingham, VT) with the excitation filters installed in a motorized filter wheel (Ludl Electronic Products, Hawthorne, NY). Image capture was performed using Micromanager (Version 1.4).

#### Virus Sequence Data Collection and Processing

Virus sequences and genome annotations were downloaded from NCBI (Refseq, last accessed October 2020, Brister et al. 2015). To exclude any regions that may be under different selective pressures due to overlapping coding regions, we employ two levels of filtering. First, using the genomic coordinates from the annotation files, we identified coding sequences that completely lie within another. For these cases, we exclude both the internal and the surrounding coding sequence. Although this an extremely conservative approach, we lose only 4.97% of all sequences (17,478/351,547). Second, we consider noncomplete overlapping coding sequences of neighboring genes. For these cases, the coordinates of each overlapping neighbor were adjusted such that the overlapping portion of each gene was removed. In all cases, coordinates were adjusted by increments of 3 nucleotides to retain the correct reading frame and codon composition in the remaining nonoverlapping sequence. As the focus of this study is on viruses that are able to infect vertebrates, we filtered the sequences further according to the host annotation from the ICTV Virus Metadata Repository (VMR) (version May 1, 2020; Walker et al. 2019). Only sequences of species annotated with a “vertebrate” or “vertebrate, invertebrate” host according to the VMR were retained. Due to ambiguity in virus genome naming of NCBI genomes (e.g., due to changes in nomenclature/taxonomy over the years), as well as typographical errors in the VMR file as well as genome files, some manual curation was conducted to retain as many viral species as possible. Furthermore, only unique sequences were retained and cds annotated as “hypothetical” were removed. This leaves 19,625 unique sequences of 1,520 virus species. Information on genome composition were taken from ICTV, while information on the replication compartment were retrieved from ViralZone (Hulo et al. 2011). For cases in which the replication compartment is not known, no compartment was assigned (NA). A list of human-infecting viruses was retrieved from virus–host DB (https://www.genome.jp/virushostdb/; accessed December 2020; (Mihara et al. 2016)). This list contained 1,360 unique virus names, corresponding to 301 unique virus species found within our curated database.

#### Calculating Sequence Parameters

To calculate dinucleotide enrichment (e.g., CpG, UpA), we take the frequency of each dinucleotide pair within the sequence divided by the product of frequencies of each individual nucleotide within the pair. The codon adaptation index (CAI) was calculated using EMBOSS version 6.6.0 and a list of 192 highly expressed human genes. The effective number of codons (ENC) was calculated using CodonW (Peden JF: Analysis of Codon Usage. University of Nottingham; 1999). To calculate ESE enrichment, the INT3 set of Exonic Splicing Enhancer motifs was downloaded from the supplement of Caceres and Hurst (2013). INT3 is composed of ESE motifs that appear in at least three of the RESCUE, Ke400, ESR, and PESE data sets, and therefore has a low false-positive rate. In order to compute a measure of enrichment of ESEs for each of the viral CDSs in our analysis, the expected number for each ESE motif was calculated from 1,000 random simulations, taking the gene structure of each sequence into account. Each simulation consisted of randomizing the order of the codons in each CDS and counting the presence of each motif along with a 6 base pairs sliding window. To obtain an overall ESEs enrichment score per CDS, the total number of observations of all INT3 motifs was added up and then compared to the expected total number of motifs from the aforementioned simulants using a Z-score. Enrichment Z-scores of the N6-methyladenosine (m6A) consensus motif, DRACH (D = A, G or U; H = A, C or U) (Linder et al. 2015), as well as overall codon pairs, were calculated in the same way.

Homo sapiens CDSs were downloaded from Ensembl release 102 (Yates et al. 2020). Sequences were filtered for the presence of a start and stop codon, as well as sequence length being a multiple of 3. Only the longest transcript isoform for each gene was used in further calculations (*n* = 20,075).

## Data Availability

The data underlying this article will be shared on reasonable request to the corresponding author.

## Supplementary Material

Supplementary data are available at *Genome Biology and Evolution* online.

## Supplementary Material

evab106_Supplementary_Data
