# Supplementary material for: Transcription, mRNA Export, and Immune Evasion Shape the Codon Usage of Viruses
Source: Genome Biol Evol. 2021 May 14;13(9):evab106. doi: 10.1093/gbe/evab106 (PMC8410142; doi:10.1093/gbe/evab106)
Supplement: evab106_Supplementary_Data [file evab106_supplementary_data.zip › Supplementary Legends_GBE_201251.docx]

**Supplementary Legends**

**Fig. S1: Comparison of sequence-derived parameters of vertebrate-infecting virus genes**, **related to Figure 2.** Measures of codon usage bias were calculated for all virus genes and grouped by Family. Boxplot representation of the variation in sequence features within each of 41 virus families. For comparison, the same parameters were calculated for human coding genes (top-most white boxes).

**Fig. S2: Sequence-feature variation in viruses within the family *Herpesviridae***, **related to Figure 2.** Density plots of sequence features calculated for all virus genes per species within the family *Herpesviridae*. Species were arranged and colour-coded according to their subfamily: Alphaherpesvirinae (blue; 34 species, n = 2337), Betaherpesvirinae (green; 19 species, n = 1968) and Gammaherpesvirinae (yellow; 18 species, n = 877). The order of species on the y-axis is identical in each panel and is determined by the median GC3 for each species within their respective subfamily.

**Fig. S3: Variation in sequence-derived parameters depending on genome properties**, **related to Figure 2.** Violin representation of sequence feature variation between species, grouped either by (A) linear and circular genomes, or (B) monopartite and segmented genomes.

**Fig. S4: Variation in sequence features depending on virus replication compartment**, **related to Figure 2.** Violin representation of sequence feature variation between species depending on their replication compartment (nucleus or cytoplasm). GC content variation between all (left panel; p = 2.2 x 10^-16^), dsDNA (middle; p = 3.37 x 10^-12^) and ssRNA(-) viruses (right; p = 2.45 x10^-12^) depending on replication compartment. (B) Observed over expected enrichment of all 16 dinucleotide pairs in virus genes, stratified by replication compartment. (C) Correlations between CpG and UpA enrichment in nuclear (left panel; not significant) or cytoplasmic (right panel; r = 0.32, p = 5.6 x 10^-19^) replicating viruses.

**Fig. S5: Fluorescence measurements of transiently transfected HeLa cells expressing GFP using different expression systems, related to Figure 4.** GFP expression in HeLa cells was measured 24h post-transfection by Fluorescence-activated cell sorting (FACS). GFP was transcribed by either endogenous RNA polymerase II (RNAPII), cytoplasmic (T7) or nuclear T7 polymerase (NLS-T7). Background expression of GFP in IRES-GFP single-transfection controls are shown in yellow bars, next expression from cells co-transfected with IRES-GFP and either cytoplasmic (green) or nuclear (blue) T7 polymerase constructs. Each data point represents the mean of 3 replicates -/+ SEM.

**Supplementary Table 1: Pairwise comparison between virus and human coding genes, related to Figure 1.** Table of p-values calculated from pairwise comparisons of sequence features of virus families with human genes. Cells containing p-values<0.05 are coloured in green.

**Supplementary Table 2: Sequences, sequence properties and FACS data of the 10 GFP variants used in transfection experiments, related to Figure 4.**

**Supplementary Table 3: Sequences of T7 polymerase and NLS-T7 polymerase used in this study.**

**Supplementary Table 4: Curated virus database used in this study, related to Figures 1-4 and S1-4.**
