# Supplementary figures and images for: Transcription, mRNA Export, and Immune Evasion Shape the Codon Usage of Viruses
Source: Genome Biol Evol. 2021 May 14;13(9):evab106. doi: 10.1093/gbe/evab106 (PMC8410142; doi:10.1093/gbe/evab106)

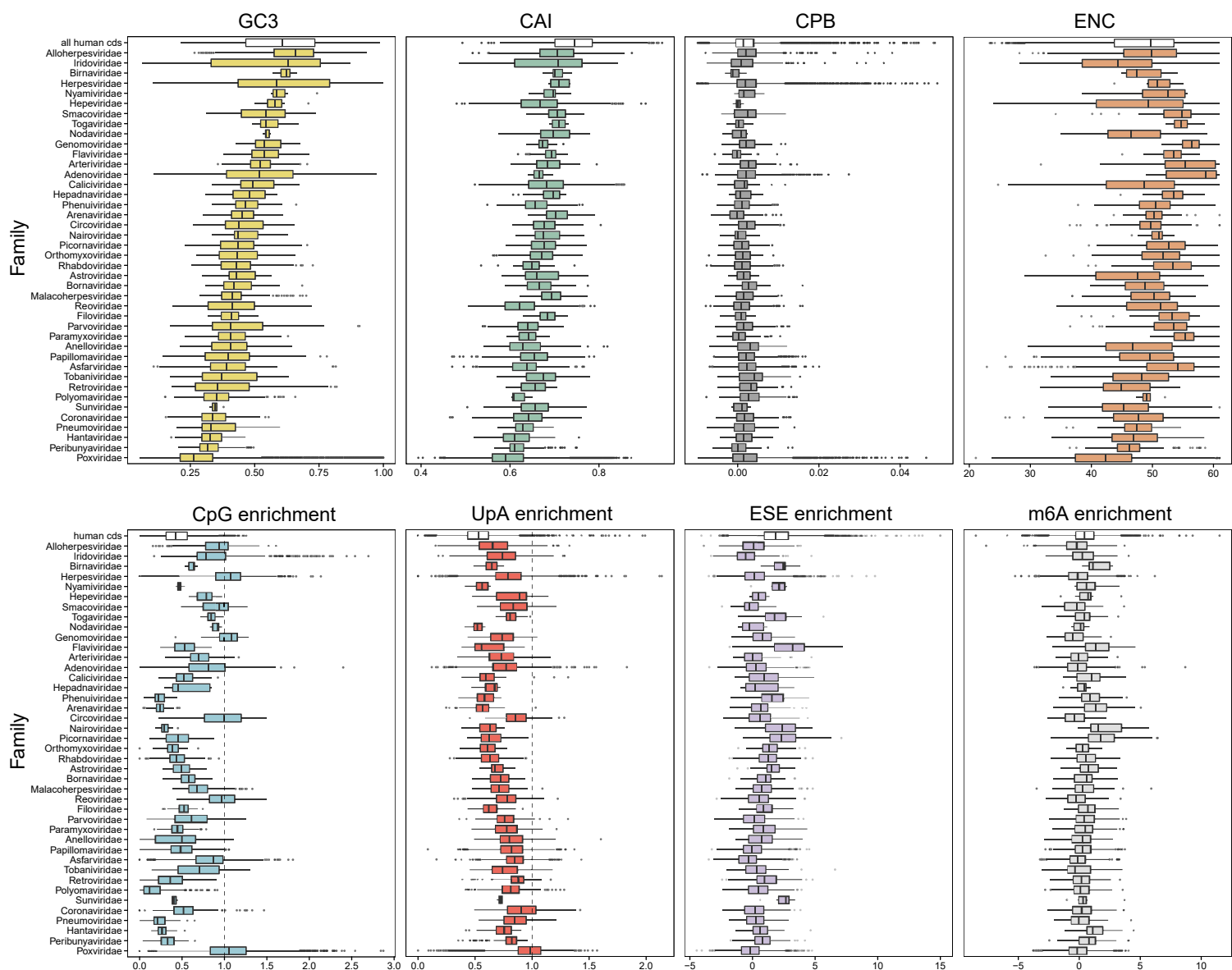

Fig. S1

Supplement: evab106_Supplementary_Data [file evab106_supplementary_data.zip › Figure S1.pdf]

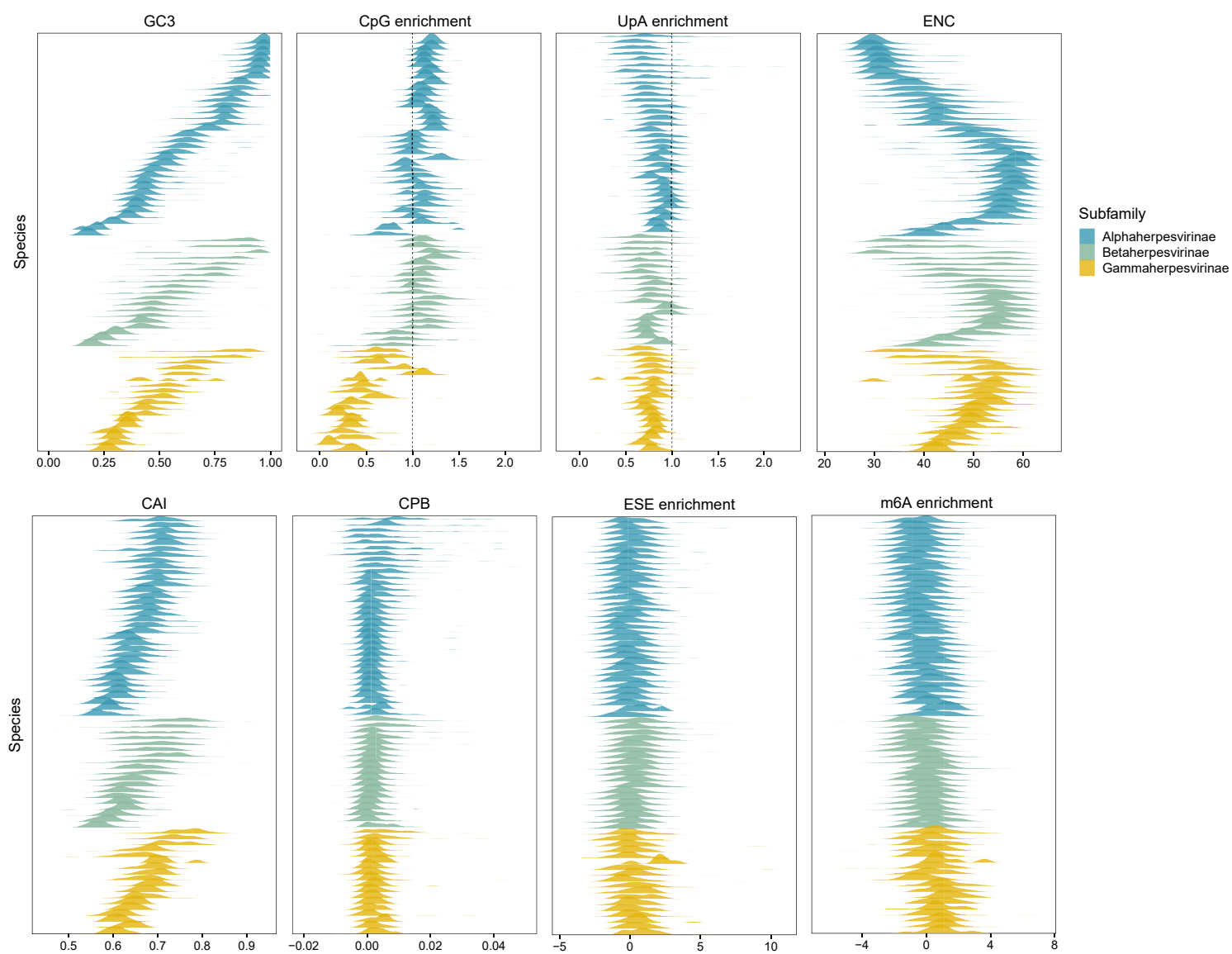

Fig. S2

Supplement: evab106_Supplementary_Data [file evab106_supplementary_data.zip › Figure S2.pdf]

A

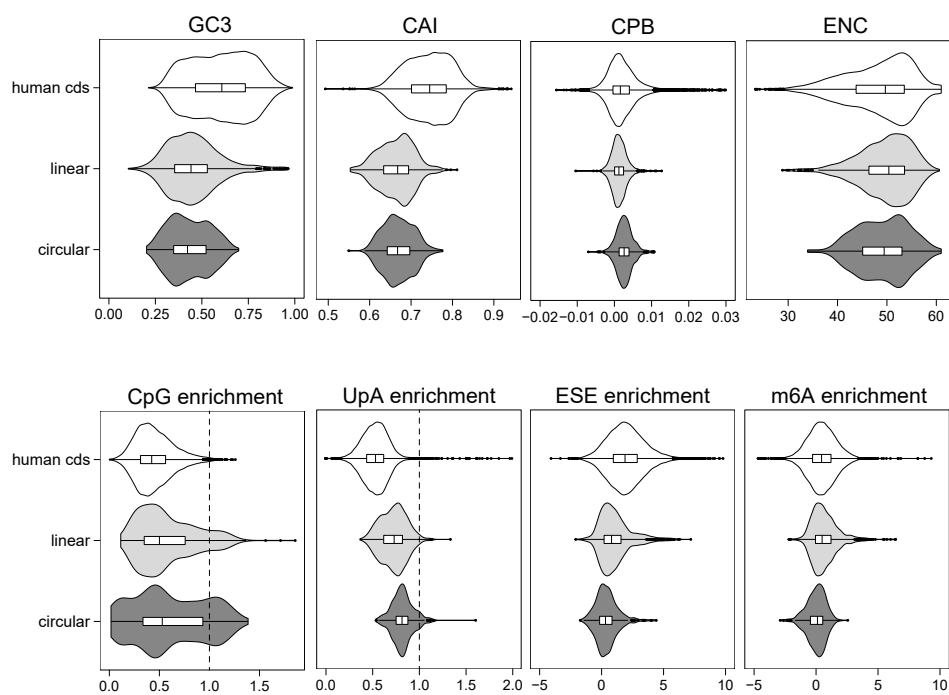

B

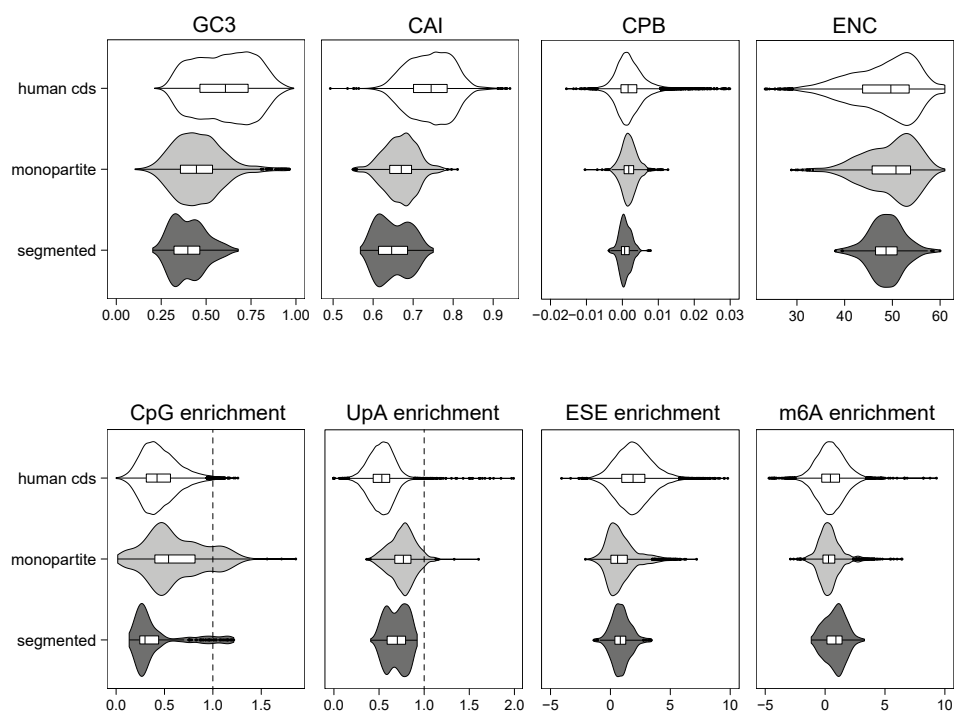

Fig. S3

Supplement: evab106_Supplementary_Data [file evab106_supplementary_data.zip › Figure S3.pdf]

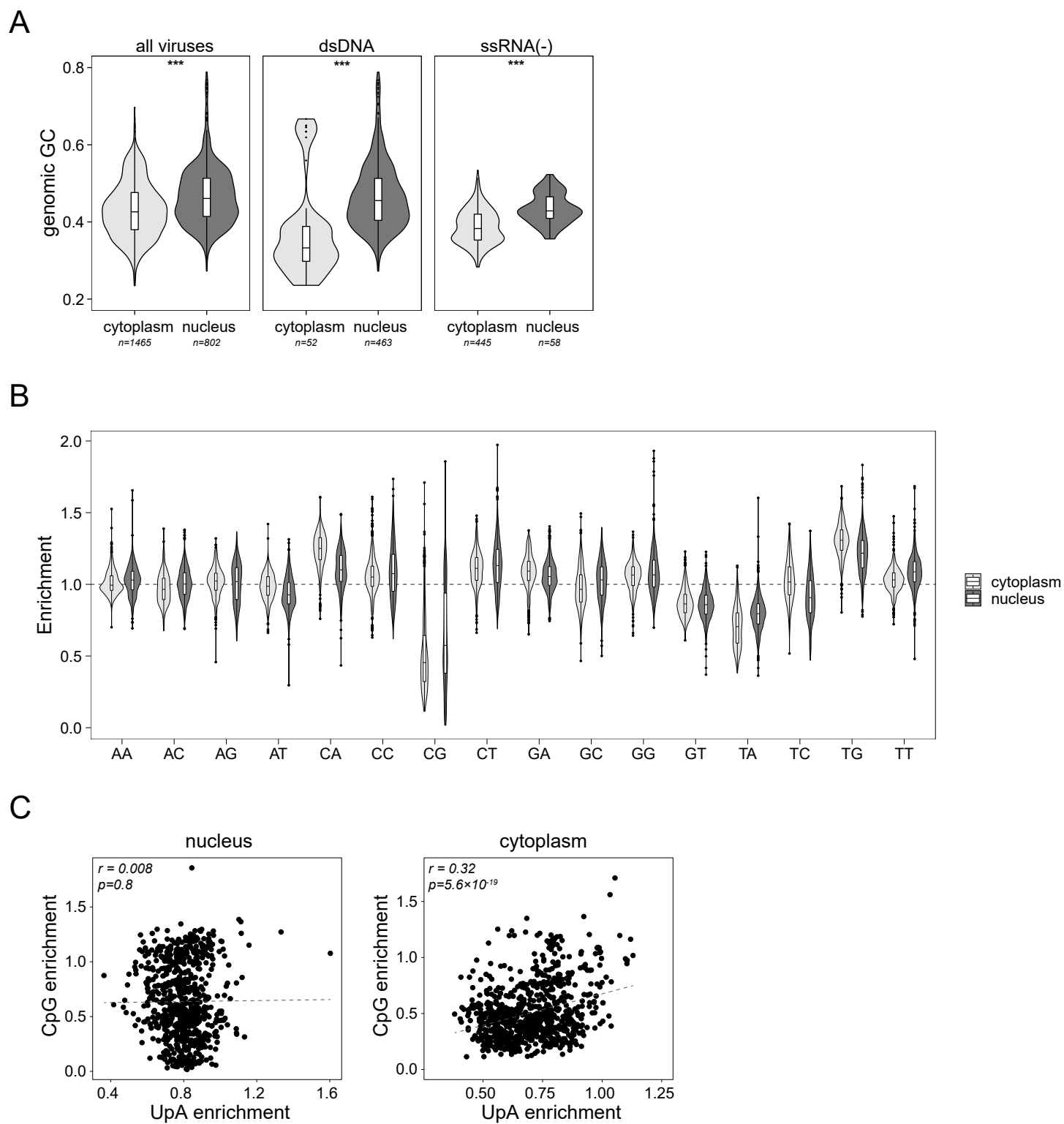

Fig. S4

Supplement: evab106_Supplementary_Data [file evab106_supplementary_data.zip › Figure S4.pdf]

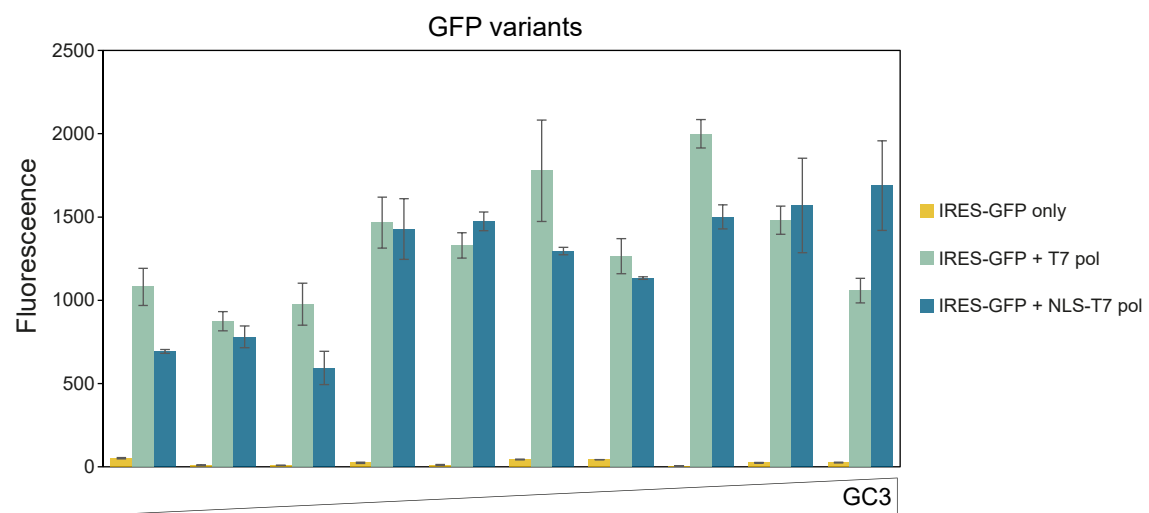

Fig. S5

Supplement: evab106_Supplementary_Data [file evab106_supplementary_data.zip › Figure S5.pdf]
